# Influence of nutritional status, laboratory parameters and dietary patterns upon urinary acid excretion in calcium stone formers.

**DOI:** 10.1590/2175-8239-JBN-3814

**Published:** 2018-04-26

**Authors:** Carolini Zanette Warmling Tessaro, Christiane Ishikawa Ramos, Ita Pfeferman Heilberg

**Affiliations:** 1 Escola Paulista de Medicina, Universidade Federal de São Paulo, São Paulo, SP, Brasil.

**Keywords:** Obesity, Obesity, Abdominal, Nephrolithiasis, Food Habits, obesidade, obesidade abdominal, nefrolitíase, dietoterapia

## Abstract

**Introduction:**

Obesity and Metabolic Syndrome (MS) are associated with low urinary pH and represent risk factors for nephrolithiasis, especially composed by uric acid. Acidogenic diets may also contribute to a reduction of urinary pH. Propensity for calcium oxalate precipitation has been shown to be higher with increasing features of the MS.

**Objective:**

A retrospective evaluation of anthropometric and body composition parameters, MS criteria and the dietary patterns of overweight and obese calcium stone formers and their impact upon urinary pH and other lithogenic parameters was performed.

**Methods:**

Data regarding anthropometry, body composition, serum and urinary parameters and 3-days dietary records were obtained from medical records of 102(34M/68F) calcium stone formers.

**Results:**

A negative correlation was found between urinary pH, waist circumference and serum uric acid levels (males). The endogenous production of organic acids (OA) was positively correlated with triglycerides levels and number of features of MS (males), and with glucose, uric acid and triglycerides serum levels, and number of features of MS (females). No significant correlations were detected between Net Acid Excretion (NAE) or Potential Renal Acid Load of the diet with any of the assessed parameters. A multivariate analysis showed a negative association between OA and urinary pH.

**Conclusion:**

The endogenous production of OA and not an acidogenic diet were found to be independently predictive factors for lower urinary pH levels in calcium stone formers. Hypercalciuric and/or hyperuricosuric patients presented higher OA levels and lower levels of urinary pH.

## RESUMO

**Introdução:**

A obesidade e a Síndrome Metabólica (SM) se associam a pH urinário ácido e representam fatores de risco para litíase renal, especialmente a úrica. Dietas acidogênicas também podem contribuir para a redução do pH urinário. Já foi demonstrado maior risco de precipitação de oxalato de cálcio em proporção aos critérios de SM.

**Objetivo:**

Avaliar retrospectivamente o impacto de parâmetros antropométricos, composição corporal, critérios de SM e padrão alimentar sobre o pH urinário e outros parâmetros litogênicos em pacientes com sobrepeso e obesos com litíase cálcica.

**Métodos:**

Foram coletados dados de antropometria, composição corporal, exames séricos e urinários, e registros alimentares (3 dias) de 102 (34M/68F) pacientes com litíase cálcica.

**Resultados:**

O pH urinário se correlacionou negativamente com a circunferência da cintura e ácido úrico sérico (homens). A produção endógena de ácidos orgânicos (AO) se correlacionou positivamente com os triglicérides séricos e o número de critérios de SM (homens), e com glicemia, ácido úrico, triglicérides e número de critérios para SM (mulheres). Não se observaram correlações significantes entre a excreção renal líquida de ácidos (NAE) e o potencial de carga ácida renal (PRAL) da dieta com nenhum dos parâmetros avaliados. Na análise de regressão multivariada, os AO apresentaram associação negativa significante com o pH urinário.

**Conclusão:**

A produção endógena de AO, e não um padrão de dieta acidogênica, foi o fator determinante independente para menores níveis de pH urinário em pacientes com litíase cálcica. Pacientes com hipercalciúria e/ou hiperuricosúria apresentaram maiores valores de AO e menores de pH urinário.

## INTRODUCTION

Metabolic Syndrome (MS) and obesity have been reported as risk factors for renal diseases, including nephrolithiasis.^1^^-^ 3 Individuals with MS tend to have a more acidic urinary pH, which is considered the most important factor in the precipitation of uric acid crystals.^3^^-^ 8 Likewise, weight and BMI are also inversely associated with urinary pH.^3^ The mechanisms by which obesity and metabolic syndrome lead to a reduction in urinary pH and, consequently, an increased risk of uric lithiasis appear to be mediated by insulin resistance, lower ammonium excretion, and H^+^ ions buffering.^8^

Preliminary studies have demonstrated an inverse correlation between body weight and net renal acid excretion (NAE) with urinary pH in uric acid stone formers. However, although 80% of renal stones are made up of calcium salts, especially calcium oxalate (CaOx),^10^^,^ 11 there are few studies that explore the relationship between MS and individuals with these types of stones. Sakhaee et al^12^ reported that the likelihood of calcium oxalate precipitation in healthy subjects was higher, but not independently associated with the number of criteria for MS.

On the other hand, the ingestion of an acidogenic diet, rich in animal protein, may also contribute to the urinary pH reduction^13^^,^ 14 due to the generation of protons during the oxidation of sulfur radicals, present in animal protein, to sulfate.^15^ The aim of our study was to evaluate the impact of anthropometric parameters and body composition, metabolic syndrome criteria and the influence of dietary patterns on urinary pH and other lithogenic parameters in overweight and obese patients with calcium lithiasis.

## PATIENTS AND METHODS

This retrospective study was based on the review of 150 medical records of patients seen at the Renal Lithiasis Outpatient Clinic of the Federal University of São Paulo (UNIFESP), between 2000 and 2008. The diagnosis of renal lithiasis had been made based on the presence of renal colic with hematuria and spontaneous elimination and/or surgical removal of the stone (s) and/or evidence of it on ultrasound imaging and plain radiography. The exclusion criteria were: age < 18 years, recurrent urinary tract infection, renal tubular acidosis, nephrocalcinosis, hyperparathyroidism, malignant diseases, chronic kidney disease (defined as estimated glomerular filtration rate < 60 mL/min/1.73 m^2^), patients without plain abdominal x-ray showing radio-opaque stones or patients who had crystallographic analysis revealing pure uric acid stones. The Medical Ethics and Research Committee of UNIFESP approved the study and all patients signed an informed consent form. The data collected included anthropometry, food intake through the 3-day food registry and serum and urinary biochemical tests. The anthropometric measures used were the Body Mass Index (BMI) and Waist Circumference (WC) of all patients. Body composition parameters were obtained from 62 patients who had undergone bioimpedance analysis (BIA) which is based on the differences in lean tissue electrical conductivity (high, due to the large amount of water and electrolytes) compared to fat and bones (low), being measured by means of distal and proximal electrodes after the passage of an imperceptible electric current, generating resistance and reactance vectors. The test is usually performed after the patient fasts for at least 4 hours, with an empty bladder, without having ingested alcohol or practiced physical activity in the last 24 hours, without any metal or prosthesis, and outside the menstrual period. The patients were classified according to BMI into: eutrophic (< 25kg/m^2^), overweight (25-29.9kg/m^2^) and obese (≥ 30kg/m^2^). The average consumption of energy, macro and micronutrients, previously calculated using the "Dietpro - version 6.0" (Federal University of Viçosa, Minas Gerais) software was recorded from the results of the 3-day food surveys. Protein, phosphorus, potassium, magnesium and calcium intake calculations were used to estimate the potential renal acid load (PRAL) according to Remer & Manz formula^16^ using the PRAL (mEq/d) = 0.49 x Protein (g/d) + 0.037 x Phosphorus (mg/d) - 0.021 x Potassium (mg/d) - 0.026 x Magnesium (mg/d) - 0.013 x Calcium (mg/d). The endogenous production of organic acids (AO) calculation was obtained by the formula proposed by Berkemeyer and Remer: 19 OA (mEq/d) = body surface area (m^2^)*x41/1.73.* Body surface area (m^2^) = 0.007184 x height (cm)^0.725^ x weight (kg)^0.425^. The net renal acid excretion (NAE) corresponding to the net body acid production,^17^^,^ 18 can be measured in the 24-hour urine by means of the difference between the sum of the titratable acidity (TA) and the ammonium excretion with the excreted bicarbonate (NAE: TA + NH_4_ - HCO_3_). Since such parameters were unavailable in the present study, the NAE was estimated by the sum of the endogenous production of OA^19^ and the PRAL obtained from the dietary data,^20^ using the formula: NAE = PRAL + OA.^16^ The biochemical evaluation included serum creatinine, urea, sodium, calcium, uric acid, total cholesterol and fractions, triglycerides, glycemia and insulin. The HOMA-IR index, which evaluates insulin resistance, was calculated using the formula: insulin_fasting_ (µU/ml) x glicemia_fasting_ (mmol/L)/22.5. The 24-hour urinary parameters used in the study were volume, calcium, sodium, citrate, uric acid, oxalate and pH.

Creatinine was determined according to the modified Jaffe's reaction, by an isotope dilution mass spectrometry (ID-MS) traceable method. Serum and urinary calcium were determined by an colorimetric method; serum and urinary uric acid, citrate, oxalate, glucose and triglycerides by an automated enzymatic method; total cholesterol and HDL by an enzymatic method; LDL was calculated using the Friedewald Equation; insulin by chemiluminescence; urinary citrate by UV/VIS spectrophotometry; and serum and urinary sodium by ion-selective electrode. All of these biochemical parameters were measured in a Beckman Clinical Chemistry Analyzer (AU480-America Inc., Pennsylvania, USA). Urine pH was measured by a pH meter (Micronal, São Paulo, Brazil). Hypercalciuria was considered when urinary calcium excretion was ≥ 250 or 300 mg/24 h (for women and men, respectively), in the presence of normocalcemia; hyperuricosuria by urinary uric acid > 750 or 800 mg/24h (for women and men, respectively); hypocitraturia by urinary citrate < 320 mg/24h and hyperoxaluria by values of urinary oxalate greater than 45 mg/24h.

### STATISTICAL ANALYSIS

Parametric or non-parametric analyses were used according to the nature of the variables evaluated by the normality test. The Chi-square or the Fisher's exact test was used to compare the categorical variables between the tertiles. The Kruskal-Wallis test was used to compare the numerical variables among the tertiles. Spearman's correlation coefficient was used to evaluate the correlation between anthropometric, serum and urinary parameters, such as pH, PRAL, OA and NAE. Univariate and multivariate linear regression analyses (Stepwise) were used to evaluate factors related to urinary pH. The level of significance was set at *p* < 0.05. The statistical analyses were carried out using the SAS System for Windows (Statistical Analysis System), version 9.2 (SAS Institute Inc., 2002-2008, Cary, NC, USA).

## RESULTS

Of the total number of records reviewed, 102 patients with calcium stones (34M/68F, 46 ± 12 years) who met the inclusion criteria were selected for the study, of whom 92 had a plain radiography exam showing radiopaque stone (s), and 10 presented crystallographic analyses revealing compositions of oxalate and/or calcium phosphate. Forty-two (41.1%) patients were hypertensive, of whom 5 (4.9%) were eutrophic, 15 (14.7%) were overweight and 22 (21.5%) were obese. Thirty-three patients (32.3%) had MS, 18 of them (17.6%) with 3 criteria, 10 (9.8%) with 4 criteria and 5 (4.9%) had 5 criteria. Only 9 (8.7%) were diabetic, of whom 2 (1.9%) were eutrophic, 1 (0.9%) was overweight and 6 (5.8%) were obese. The mean BMI of the participants was 29.5 ± 5.3 kg/m^2^. According to the BIA, the mean percentage of body fat and lean mass were 26 ± 6% and 31 ± 8%, among men, and 36 ± 7% and 42 ± 7% among women, respectively. The mean WC for men was 99 ± 6 cm and 97 ± 4 cm for women (data not shown in the table). The distribution of serum and urinary biochemistry and anthropometric values, according to BMI categorization, is shown in Table 1. Among males, 9 (26.4%) were eutrophic, 11 (32.3%) were overweight and 14 were ( 41.1%) obese. Among women, 14 (20.5%) were eutrophic, 25 (36.7%) were overweight and 29 (42.6%) were obese. Of the total number of patients, only 3 (1M/2F) were grade III obese (BMI ≥ 40kg/m^2^). The production of OA was significantly higher among obese and overweight patients when compared to their eutrophic counterparts (48 ± 5, 44 ± 3 *versus* 40 ± 2 *p* = < 0.001 and 43 ± 2, 41 ± 2 *versus* 37 ± 2 *p* = < 0.001) for men and women, respectively (data not shown in the table). Compared to eutrophic men, their obese and overweight counterparts did not present significant differences in PRAL (37 ± 4, 16 ± 4 *versus* 27 ± 5 *p* = 0.603), and the same was true for women (34 ± 5, 17 ± 4 *vs*. 27 ± 4 *p* = 0.785) (data not shown in the table). Compared to normal men, serum HDL was significantly lower among obese men. Urinary creatinine was significantly higher in obese and overweight *versus* eutrophic patients. Obese and overweight women had significantly higher fasting glycemia, HOMA-IR, serum and urinary uric acid, when compared to their eutrophic counterparts. There was no statistical difference in urinary pH or NAE between BMI categories in both genders. Table 2 depicts correlations between pH, PRAL, OA and NAE with anthropometric, serum and urinary parameters. We did not calculate the correlation between OA production and the anthropometric parameters, since the first one is calculated from body surface. Urinary pH correlated negatively with WC and serum uric acid among men, but not among women. OA correlated with serum triglyceride levels and the number of criteria for MS among men, and serum levels of glycemia, uric acid, triglycerides and the number of criteria for MS among women. There were no significant correlations between pH, PRAL, NAE and OA with any of the urinary parameters. Table 3 presents the averages of urinary pH, anthropometric parameters, NAE, PRAL and OA according to the presence or absence of urinary metabolic changes alone or in combination. Urinary pH was significantly lower and OA production was significantly higher among patients with hypercalciuria, compared to those without the disorder. There was no statistical difference between the different parameters regarding the presence of hypocitraturia. On the other hand, urinary pH was significantly lower and the OA and BMI significantly higher in the presence of hyperuricosuria, alone or in combination. The urinary oxalate dosage was only available in 20% of the sample, and among these, no patient presented hyperoxaluria, which is why a subanalysis of patients with and without the disorder was not performed. Finally, determinants of urinary pH assessed by regression analysis are shown in Table 4. In the univariate analysis, serum levels of triglycerides, uric acid and OA showed a significant negative association with urinary pH, and only OA showed a significant negative association with urinary pH in the multivariate analysis, remaining in the model as an independent determinant factor.

**Table 1:** Urinary and serum anthropometric parameters according their classification as per the Body Mass Index

		MEN				WOMEN		
	Eutrophic (n = 9)	Overweight (n = 11)	Obese (n = 14)	*p*	Eutrophic (n = 14)	Overweight (n = 25)	Obese (n = 29)	*p*
*ANTHROPOMETRIC*								
WC	91 ± 3	97 ± 6	102 ± 6	0.654	89 ± 3	94 ± 4	103 ± 7	0.459
BF (%)	25 ± 5	31 ± 8	39 ± 11	0.973	27 ± 7	30 ± 5	34 ± 7	0.736
LM (%)	30 ± 8	41 ± 9	35 ± 8	0.539	30 ± 6	38 ± 6	38 ± 9	0.526
*SERUM*								
Fasting glucose	105 ± 30	96 ± 34	105 ± 29	0.767	85 ± 17	93 ± 19^a^	102 ± 35^a^	0.028
HOMA-IR	3.2 ± 2.0	2.4 ± 1.4	3.9 ± 2.0	0.503	1.4 ± 0.6	2.6 ± 0.5^a^	3.3 ± 1.4^a^	0.020
Calcium	9.4 ± 0.3	9.6 ± 0.3	9.4 ± 0.3	0.639	8.6 ± 2.2	9.2 ± 0.4	9.4 ± 0.4	0.132
Uric acid	6.5 ± 1.3	6.7 ± 1.8	7.0 ± 1.4	0.558	4.0 ± 1.2	5.2 ± 1.2^a^	5.1 ± 0.9^a^	0.008
HDL	51 ± 11	43 ± 9	38 ± 7^a^	0.035	53 ± 12	51 ± 14	49 ± 12	0.562
Triglycerides	133 ± 60	183 ± 24	248 ± 65	0.156	101 ± 24	132 ± 54	152 ± 76	0.077
*URINARY*								
Volume	2203 ± 681	1924 ± 628	2355 ± 826	0.414	1780 ± 550	1846 ± 710	1926 ± 762	0.962
Creatinine	1414 ± 165	1778 ± 339^a^	1917 ± 376^a^	0.002	1122 ± 243	1284 ± 274	1258 ± 303	0.331
Calcium	222 ± 113	200 ± 92	233 ± 140	0.898	203 ± 100	197 ± 119	199 ± 95	0.864
Uric acid	655 ± 214	739 ± 323	828 ± 271	0.272	397 ± 67	583 ± 96^a^	597 ± 145^a^	< 0.0001
Citrate	262 ± 23	483 ± 30	457 ± 45	0.163	318 ± 84	344 ± 188	307 ± 211	0.940
Sodium	218 ± 87	223 ± 65	282 ± 90	0.189	164 ± 54	211 ± 85	217 ± 81	0.096
Oxalate	31 ± 3	37 ± 9	41 ± 7	0.451	28 ± 3	28 ± 5	36 ± 5	0.398
pH	6.3 ± 0.7	6.1 ± 0.5	5.9 ± 0.8	0.251	6.1 ± 0.6	6.1 ± 0.7	6.1 ± 0.5	0.929
NAE	67 ± 18	61 ± 21	85 ± 32	0.375	65 ± 18	58 ± 22	79 ± 25	0.591

Mean ± SD. WC: Waist Circumference; BF: Body Fat; LM: Lean Mass; NAE: Net Acid Excretion.

a significance vs. Eutrophic.

**Table 2:** Correlation (r) between anthropometric, serum and urinary parameters with pH, PRAL, NAE & OA

	MEN				WOMEN			
	pH	PRAL	NAE	AO	pH	PRAL	NAE	AO
*ANTHROPOMÉTRICS*								
BMI	-0.23	0.06	0.14	-	-0.04	0.04	0.12	-
WC	-0.38^a^	-0.04	0.04	-	-0.11	-0.04	0.01	-
BF (%)	-0.23	0.20	0.28	-	0.00	0.11	0.12	-
*SERUM*								
Creatinine	-0.02	-0.12	-0.14	-0.01	0.05	0.10	0.07	-0.06
Glucose	-0.03	-0.20	-0.20	-0.01	-0.01	0.00	0.03	0.25^a^
HOMA-IR	-0.36	0.17	0.18	0.13	0.07	0.24	-0.15	0.26
Calcium	-0.05	-0.17	-0.17	-0.03	0.08	0.08	0.10	-0.02
Uric Acid	-0.43b	-0.11	-0.09	0.12	-0.12	0.00	0.02	0.34^b^
HDL	0.06	0.19	0.16	-0.24	-0.08	0.23	0.21	-0.08
Triglycerides	-0.34	0.05	0.09	0.41^a^	-0.16	-0.05	-0.03	0.28^a^
# MS criteria	-0.29	-0.07	-0.01	0.46^b^	-0.16	-0.05	0.00	0.48^c^
*URINARY*								
Uric acid	-0.21	0.03	0.07	0.32	-0.02	0.06	0.01	0.21
Calcium	-0.04	-0.02	-0.08	0.05	-0.05	-0.12	-0.05	0.11
Citrate	0.69	0.11	0.13	0.17	0.06	0.05	0.07	0.11
Sodium	-0.54	-0.02	0.12	0.21	-0.42	-0.01	0.03	0.21
Oxalate	0.32	0.11	0.19	0.21	0.16	0.13	0.17	0.12

BMI: Body Mass Index; WC: Waist Circumference; BF: Body Fat; PRAL: Potential Renal Acid Load; OA: Organic Acid; NAE: Net Acid Excretion;

a p < 0.05;

b *p* < 0.01;

c *p* < 0.001.

**Table 3:** Association between urinary pH, NAE, PRAL, OA and anthropometric parameters with the presence of metabolic disorders

	HYPERCALCIURIA			HYPOCITRATURIA			HYPERURICOSURIA		
	NÃO	SIM	p	NÃO	SIM	p	NÃO	SIM	p
	(n=35)	(n=67)		(n=58)	(n=44)		(n=26)	(n=76)	
pH	6.2 ± 0.6	5.9 ± 0.6	0,038	6.1 ± 0.7	6.1 ± 0.5	0,412	6.1 ± 0.7	5.8 ± 0.5	0,017
PRAL	32 ± 5	17 ± 4	0,260	19 ± 4	37 ± 5	0,313	27 ± 4	28 ± 4	0,460
OA	42 ± 3	44 ± 4	0,025	42 ± 3	43 ± 4	0,395	42 ± 3	45 ± 4	0,011
NAE	74 ± 41	62 ± 24	0,322	62 ± 41	80 ± 35	0,236	69 ± 29	73 ± 25	0,265
WC	96 ± 10	101 ± 13	0,079	96 ± 10	99 ± 13	0,579	95 ± 10	103 ± 12	0,006
BMI	29 ± 5	30 ± 5	0,575	29 ± 5	29 ± 5	0,668	28 ± 5	31 ± 4	0,021

WC: Waist Circumference; BMI: Body Mass Index; PRAL: Potential Renal Acid Load; OA: Organic Acid; NAE: Net Acid Excretion.

**Table 4:** Univariate and multivariate linear regression analysis for urinary pH

	UNIVARIATE ANALYSIS *			MULTIVARIATE ANALYSIS *		
	β (EP)	R^2^	p	β (EP)	R^2^	p
WC	-3,87 (6,28)	0,003	0,53			
BMI	-0,08 (0,10)	0,007	0,38			
HBP	-10,34(5,89)	0,02	0,08			
Glycemia	-0,03 (0,11)	0,001	0,76			
Serum Uric Acid	-0,19 (0,09)	0,03	0,04			
Triglycerides	-0,24 (0,10)	0,05	0,02			
PRAL	-0,10 (0,10)	0,009	0,34			
OA	-0,23 (0,09)	0,05	0,01	-0,32(0,10)	0,10	0,002
NAE	-0,11 (0,10)	0,01	0,27			

* Gender adjusted. WC: Waist Circumference; BMI: Body Mass Index; PRAL: Potential Renal Acid Load; OA: Organic Acid; NAE: Net Acid Excretion.

## DISCUSSION

The present study aimed to evaluate the impact of anthropometric parameters and body composition, metabolic syndrome criteria and the influence of dietary patterns on urinary pH and other lithogenic parameters in overweight and obese patients with calcium lithiasis.

The main finding was that the endogenous production of organic acids (OA), estimated from anthropometric values, and not an acidogenic diet - evaluated by PRAL, constituted an independent determinant of urinary pH reduction in patients with calcium lithiasis. Patients with hypercalciuria and/or hyperuricosuria had a more acidic urinary pH and higher OA values.

Obesity or weight gain, especially when accompanied by Metabolic Syndrome, have been associated with a reduction in urinary pH,^21^^,^ 22 and a higher incidence and prevalence of renal lithiasis, especially in relation to uric acid stones.^3^^,^ 23 ^-^ 25 Meanwhile, epidemiological studies do not distinguish between the types of stones formed. From the year 2000, the first reports of more acidic urinary pH appeared in patients with severe obesity.^26^ Subsequently, Maalouf et al.,^21^ in a cohort study involving 4883 kidney stone patients, found a strong inverse and gradual correlation between levels of urinary pH and body weight, but without specifications as to the type of stone. However, although Sakhaee *et al*.^12^ found a reduction in pH in patients with MS, calcium lithiasis was not independently associated with elevated urinary saturation of calcium oxalate. Therefore, it is not possible to establish a relationship between the reduction of urinary pH and the formation of calcium stones. Finally, in a recent large longitudinal retrospective study involving 35-year-old patients with lithiasis, Xu et al.^27^ found an increase in the proportion of patients with uric acid stones from 7% to 14%, who presented higher BMI and lower pH in relation to patients with calcium oxalate stones, that represent the vast majority. However, according to these investigators, the reduced urinary pH was the best indicator of uric acid stones, but not of calcium.

Excessive consumption of animal protein has also been associated with a reduction in urinary pH in non-lithiasis individuals, with or without MS.^14^ In previous studies, although a higher prevalence of overweight was found in patients with lithiasis,^28^^-^ 30 most of them did not show higher protein intake than controls,^30^^,^ 31 except during the weekends.^28^ Recently, Ferraro et al.^32^ studied the intake of animal protein (derived from dairy products or not) and vegetable protein, and concluded that the risk of incident renal stones may vary according to the type of protein ingested. The higher intake of animal protein of non-dairy origin was associated with higher excretion of uric acid and lower urinary pH, but surprisingly not with calciuria.^32^

In the present study, involving only patients with calcium lithiasis, there was a non-statistically significant trend towards the reduction of urinary pH in overweight and eutrophic subjects (6.2 ± 0.3 and 6.1 ± 0.2 *versus* 6.0 ± 0.3 *p* = 0.51) *versus* obese individuals (BMI > 30kg/m^2^) (data not shown in the table). Due to gender-inherent differences in body composition, a subanalysis of the various nutritional and biochemical parameters was performed separately for each gender. However, even when evaluated according to gender, we found no significance in relation to the reduction of urinary pH in the obese subgroups of both genders. These results differ from those found by other investigators,^25^^,^ 26 ^,^ 33 ^-^ 35 especially regarding those who evaluated patients with a greater degree of obesity than those in the present series.^26^^,^ 33 However, unlike our sample, including only patients with calcium stones, the other studies also evaluated patients with uric acid lithiasis,^25^^,^ 34 which should have influenced the results due to the well-established relationship between uric acid and urinary pH. Shavit et al.,^25^ although finding a reduction in urinary pH, not only in obese but also in those who were overweight, also showed a higher incidence of uric acid stones, but not calcium lithiasis. Sakhaee et al.,^12^ found a reduction in urinary pH in patients with pure uric acid stones compared to calcium oxalate, but they did not find pH differences in relation to mixed stones.

Among the goals of the present study, one was to evaluate the impact of overweight or obesity and the number of criteria for MS, not only on urinary pH, but also on the 24-hour urinary composition, including renal acid excretion estimated by NAE - which was higher in the obese groups of both genders, in relation to the eutrophic, but without reaching statistical significance. This data is similar to those from Sakhaee et al.,^6^ who also did not find a statistically significant elevation of NAE in those with calcium oxalate or mixed stones, but only in patients with uric lithiasis. In the present sample, since the crystallographic analysis of the stones' composition was available in only 10% of the medical records, we considered all the radiopaque stones to be calcium lithiasis, but without discrimination in mixed compositions involving calcium phosphate or uric acid,^27^^,^ 36 with different impacts on urinary pH. In any case, the NAE did not correlate with any of the lithogenic urinary parameters. Among the other urinary parameters, we found in this series only an increase in the mean values of urinary creatinine excretion among obese men and of urinary uric acid among overweight and obese women, in relation to the eutrophic ones. Although some studies report a higher percentage of hypercalciuric individuals in the obese group,^23^^,^ 26 in the present sample there was no increase in the mean urinary calcium excretion among the obese, which is in agreement with that reported by Shavit et al.^25^ However, in this series we found a significant reduction in urinary pH, specifically among patients who presented hypercalciuria, alone or associated with other disorders, when compared to patients with normal urinary calcium and of similar BMI. In hyperuricosuric patients, alone or in association, a significantly lower mean urinary pH and significantly higher BMI and OA production were found, when compared to patients without the disorder. Interestingly, Sakhaee et al.^6^ also found high urinary uric acid in patients with calcium or mixed oxalate stones, with BMI values similar to the ones in the present study.

Considering that the diet may affect the urine's acid-base state, we used an estimate of the acid load of foods and diet in general, obtained by calculating PRAL, which provides an estimate of endogenous acid production that exceeds the level of alkali produced by certain food groups consumed daily. PRAL can be calculated from the food record itself and it is directly related to NAE. There are no normal values for PRAL, but values tending to positive indicate acid loads, while negative values translate into excess bases. Although there was a tendency for higher levels of PRAL among the obese individuals in the present study, eutrophic patients also presented numerically higher values than their overweight counterparts, resulting in the absence of statistical difference in these parameters between the subgroups of both genders. In addition, PRAL did not correlate with any of the parameters analyzed in the present study, including anthropometric and urinary biochemical ones. Taken together, our findings do not suggest that the dietary pattern has had significant influence on urinary pH. On the other hand, it cannot be ruled out that the measure of urinary sulphate,^37^ as well as the determination of ammonium excretion, titratable acidity and bicarbonaturia, which would more adequately represent the acid excretion resulting from an acidogenic diet, could reveal different results, but such parameters were not available in the medical charts for the present study. However, Maalouf et al.^21^ reported that the inverse relationship between urinary pH and body weight persisted, even after adjustment for urinary sulphate, suggesting a diet-independent mechanism, which was also found for patients with uric lithiasis, even in neutral-content diets.^5^^,^ 6 In addition, although individuals with MS had significantly higher urinary sulphate, which suggested an acidogenic diet, for any level of urinary sulphate, pH was significantly lower among individuals with MS, suggesting factors not associated with diet, interfering with urinary pH.

Regarding serum parameters related to MS among men, we noticed that the subgroup of obese individuals had lower levels of HDL - a finding similar to that of a large cohort of lithiasis evaluated by Torricelli et al.,^38^ who reported lower serum HDL levels, higher triglyceride levels, as well as lower urinary pH in patients with higher BMI. These researchers also found an association between low HDL and elevated triglycerides and formation of uric acid stones, which was not the focus of the present study. In any case, men in the present series had an inverse correlation between urinary pH and serum uric acid, and the OA correlated directly with serum triglyceride levels. Among women of the present series, we also found a positive correlation between OA and glycemia and serum levels of uric acid and triglycerides. These findings are in agreement with those from Powell et al.,^26^ who also detected higher blood glucose and serum uric acid levels in obese female subjects. In addition, OA correlated positively with the number of criteria for MS for both genders, which may increase the risk of calcium oxalate stone formation, according to Sakhaee *et al*.^12^

Regarding body composition, there was an inverse correlation between pH and WC, which reflects the visceral fat component in men, but not the percentage of total body fat. Among women, there was no correlation between pH and any body composition parameter. These findings differ from those reported by Pigna et al.,^39^ who reported a higher percentage of visceral fat related to urinary acid pH in patients of both genders. However, it is possible that the bioimpedance exam was not sensitive enough to detect statistical differences, since in this other study,^39^ DEXA, a gold standard method, for assessing body composition was used. In any case, the reason why there was no inverse correlation between pH and WC in women remains to be clarified.

In the present regression analysis, urinary pH was associated with serum uric acid, triglyceride levels, and with OA production, but it was in an independent parameter only with this last one. An inverse association between NAE and urinary pH has been described by other investigators,^14^^,^ 40 ^,^ 41 but not between urinary pH and OA. The absence of an inverse association between urinary pH and PRAL in the present series suggests that a balance between the consumption of animal protein (with greater influence on urinary pH) and the other macronutrients, such as lipids and carbohydrates, characterizes the dietary pattern of these patients without privileging any of them. Among the limitations of the present study, there are the ones related to it being a retrospective study, less availability of bioimpedance and urinary oxalate results, and lack of crystallographic stone analyses for most of the patients.

In conclusion, the present study suggests that the endogenous production of organic acids (OA) rather than an acidogenic diet was an independent determinant for lower urinary pH levels among patients with calcium lithiasis. Higher values of OA and lower pH were found in patients with hypercalciuria and/or hyperuricosuria.
